# Bell-Tawse procedure with cortical fixation using endo-button in anterior radial head dislocation: A case report

**DOI:** 10.1016/j.ijscr.2025.111080

**Published:** 2025-02-22

**Authors:** Renaldi Prasetia, Siti Zainab Bani Purwana, Azir Adityo Rahman Dibyosubroto, Yosep A. Tarong, Raden Andri Primadhi

**Affiliations:** a Departement of Orthopaedics – Traumatology, Universitas Padjadjaran, Hasan-Sadikin General Hospital, Bandung, Indonesia; b Faculty of Medicine, Universitas Padjadjaran, Hasan-Sadikin General Hospital, Bandung, Indonesia

**Keywords:** Anterior radial head dislocation, Bell Tawse, Endo-button, Monteggia fracture, Case report

## Abstract

**Introduction:**

Anterior radial head dislocations are rare in adults. Misdiagnosis to Monteggia fracture-dislocation can occur up to 28 %, which results in failed detection of the misaligned radiocapitellar joint. Anterior radial head dislocation is treatable using the Bell Tawse technique. However, reports regarding the technique are rare in adults, with no endo-button use.

**Case presentation:**

A 26-year-old male came to the orthopedic outpatient clinic with left elbow pain as his chief complaint. The affected hand was a non-dominant hand. The patient had a work-related injury where the fractures were already immediately treated with open reduction internal fixation (ORIF) 4 months prior. Radiograph images showed anterior radial head dislocation with internally fixated distal-third humerus and proximal-third ulnar fractures. The patient was diagnosed with recurrent radial head dislocation post-Monteggia facture. The patient underwent Bell Tawse with cortical fixation using an endo-button.

**Discussion:**

After 1 year, the patient felt no more pain (visual analog scale (VAS) score 0). The patient was satisfied with the one-year outcome with improved elbow function. The American Shoulder and Elbow Surgeons (ASES) score and the disabilities of the arm, shoulder, and hand (DASH) score improved.

**Conclusion:**

Endo-button hadn't been used in annular ligament reconstruction, especially the Bell Tawse procedure. In our report, we used endo-button cortical fixation to compensate for the inadequate graft length in an adult case. At the one-year follow-up, the patient had no complaints of pain and could do normal activities.

## Highlights

• Monteggia fracture-dislocations are rare in adults.
• Chronic dislocation, often due to ulnar deformity and/or annular ligament failure
• The use of endo-button helps in adding extra length to grafts.

## 1 Introduction

Radial head dislocations are rare in adults [1]. Anterior dislocations, to be precise, are even rarer, accounting for only 0 %–2.6 % of elbow dislocations [2,3]. It has been reported to happen following previous surgery [2]. The typical mechanisms involved are direct impact, axial loading, torsional, and distraction force [2,3]. Monteggia fracture-dislocation is characterized by proximal-third ulnar shaft fracture and radial head dislocation [4,5]. The fracture-dislocation has less than 2 % incidence with misdiagnosis occurring up to 28 %, one of the causes being surgeons are too focused on ulnar fracture reduction and fixation, which results in failed detection of the misaligned radiocapitellar joint. The misdiagnosis can cause neglected Monteggia fracture-dislocation and lead to deformities and dysfunction of the forearm [5].

A Monteggia fracture-dislocation can be managed with gentle traction to reduce radiocapitellar joint and ulnar fixation [6]. Surgical fixation is then indicated in recurrent elbow dislocation with Bell Tawse technique as one of the options for anterior radial head dislocation [7,8]. However, reports regarding the technique are scarce, especially in adults, and none used endo-button.

We reported a case of recurrent anterior elbow (radial head) dislocation with previously internally fixated proximal third ulna fracture (Monteggia fracture-dislocation) and previously internally fixated distal-third humerus fracture treated with modified Bell Tawse procedure using endo-button cortical fixation. Informed consent for this report was given by the patient and this work has been reported in line with the 2023 SCARE criteria [9].

## 2 Case presentation

A 26-year-old male came to the orthopedic outpatient clinic with left elbow pain as his chief complaint. The affected hand was non-dominant; his visual analog scale (VAS) was 6. The patient had a work-related injury 4 months prior, where he suffered a direct blow to the elbow from a machine with elbow extension and arm pronation. After the injury, he was diagnosed with an open distal third humeral fracture and a proximal third ulnar fracture. The patient's fractures had been treated with open reduction internal fixation (ORIF) by another orthopedic surgeon. However, his pain persisted, and his range of motion remained limited, with a prominent “click” sound during elbow flexion. The patient was then referred to our outpatient clinic for further examination and treatment.

**Fig. 1:**
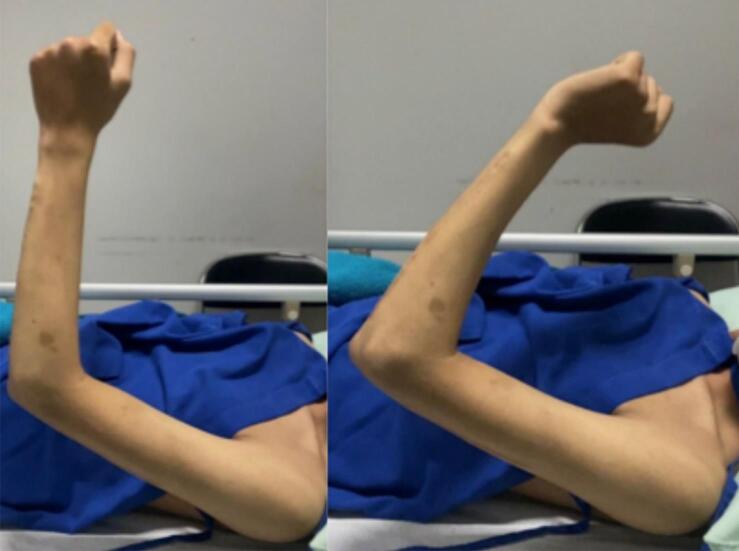
Patient's clinical presentation.

**Fig. 2:**
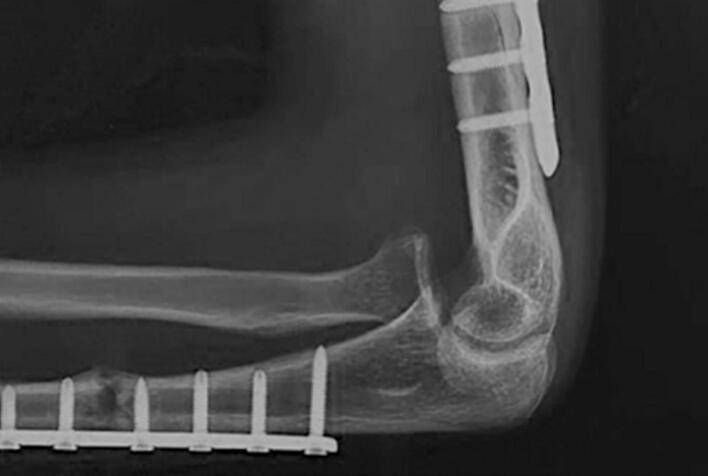
Pre-operative radiograph.

A physical examination revealed limited left elbow motion. The range of motion (ROM) of the left elbow was 100.2° in flexion and 69.5° in extension (Fig. 1). Radiograph images showed anterior radial head dislocation with internally fixated distal-third humerus and proximal-third ulnar fractures (Fig. 2). Ulnar shortening was ruled out as the cause of the dislocation. The clinical examination and x-ray confirmed proper length and alignment compared to the unaffected side. The patient was diagnosed with recurrent radial head dislocation post-Monteggia facture. The patient underwent annular ligament reconstruction with the Bell Tawse procedure.

### 2.1 Surgical technique

**Fig. 3:**
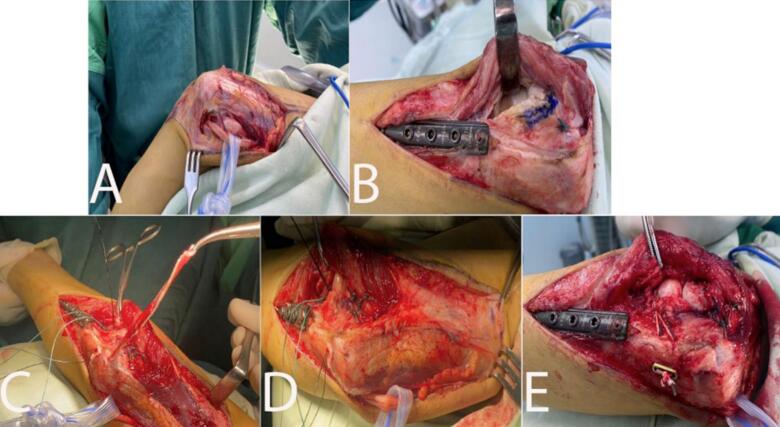
Surgical procedure. (A) Speed and Boyd Approach. (B) Exposing radiocapitulum. (C) Graft preparation. (D) Tunnel preparation. (E) Post-graft insertion, capsular repair, and endo-button fixation.

**Fig. 4:**
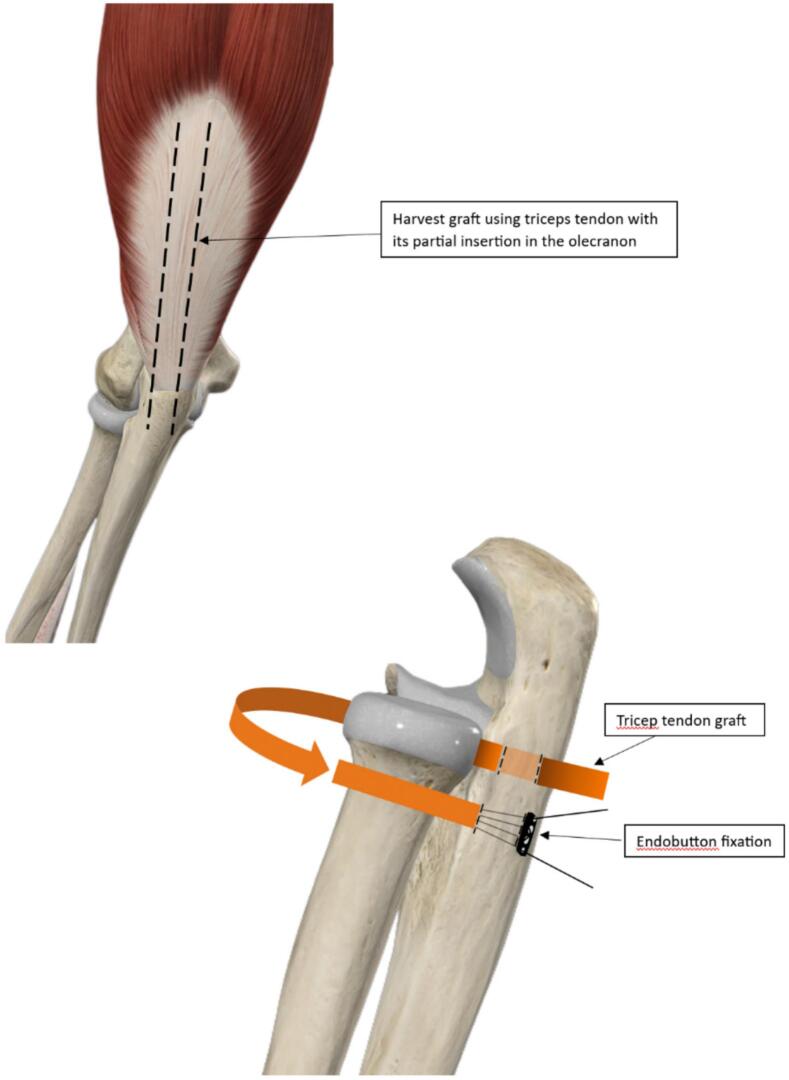
Illustration of Bell-Tawse Procedure, Fixated with Endobutton.

**Fig. 5:**
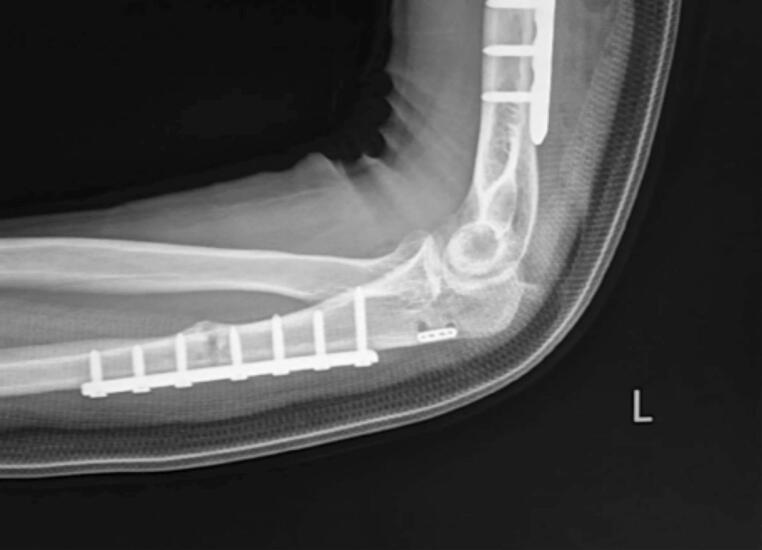
Post-operative radiograph.

The procedure was conducted under general anesthesia and performed by the senior author. The patient was positioned in a lateral decubitus position. A single incision was made with the Speed and Boyd approach for the open reduction (Fig. 3). We evacuated and protected the ulnar nerve, then exposed the radial head until the radiocapitellar joint and the lateral ulnar collateral ligament were visible. Graft preparation for annular ligament reconstruction was done using a tricep tendon that was 10 cm long and 0.5 cm wide (Bell Tawse). The radiocapitellar joint was then opened to expose the radial head. Fibrous tissues interfering with the reduction were removed. A drill hole tunnel was then made through the ulna. The graft seemed too short to be applied according to the original Bell Tawse method, where the distal end of the tricep graft was fixated through the tunnel and stitched near the insertion part [10]. To compensate for the inadequate length, the distal end of the graft was fixated with an endo-button (Smith & Nephew, Andover, MA, USA). The graft, still attached to its ulnar insertion, was inserted into the tunnel and wrapped around the radial neck. The patient then underwent dislocation reduction. The graft was then inserted into a second tunnel through the ulna with an opening in the dorsal aspect of the ulna near the articular side of the proximal radioulnar joint and fixated with an endo-button in the other end of the tunnel, which was located slightly inferomedial to the first tunnel (Fig. 4). Capsular repair was also performed. After the fixation, stability was checked to evaluate the procedure. Radiograph imaging of the left elbow was taken right after wound closure (Fig. 5). The patient's left elbow was then fixated with a cast in a 90° flexion.

### 2.2 Outcome and follow-up

**Fig. 6:**
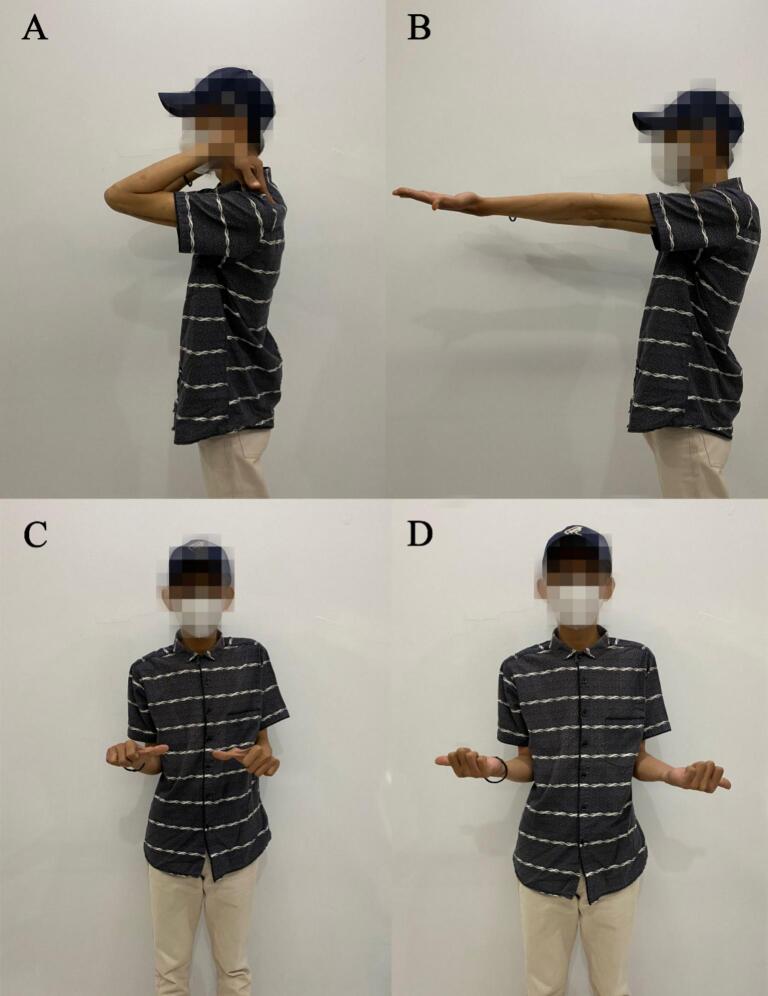
Patient's range of motion at one-year follow-up. (A) Elbow flexion. (B) Elbow extension. (C) Pronation. (D) Supination.

Full wound closure was achieved in 2 weeks. Posterior splint was applied to the elbow, proximal to the wrist joint, with the elbow placed in the neutral position. The splint was removed 3 weeks after the procedure. Rehabilitation began immediately after cast removal and passive movements were encouraged. Normal active movements were achieved after 2 months (VAS score 1). At the one-year follow-up (Fig. 6), elbow ROM was 0° in extension, 132.8° flexion, 93° in supination and 75.9° pronation (Table 1). After 1 year, the patient felt no more pain (VAS score 0). The patient was satisfied with the one-year outcome of improved elbow function, including object-lifting, daily activities, work-related movements, and recreational sporting activities. The American Shoulder and Elbow Surgeons (ASES) score improved from 28 to 100. A similar result was also found in the functional assessment using disabilities of the arm, shoulder, and hand (DASH) score (95.8–5.8).

**Table 1:** Pre- and post-operative assessments.

Assessment tool	Preoperative	1-Year follow-up
VAS	6	0
ASES score	28	100
DASH score	95.8	5.8
ROM		
a. Elbow flexion	100.2°	132.8°
b. Elbow extension	69.5°	0°
c. Supination	n/a	93°
d. Pronation	n/a	75.9°

VAS: visual analog scale; ASES: The American Shoulder and Elbow Surgeons; DASH: disabilities of the arm, shoulder, and hand; ROM: range of movements.

## 3 Discussion

Dislocation of the radial head in a Monteggia fracture if left untreated, can cause elbow pain and decreased motion, as seen in our patient. Neurologic problems or valgus deformity can also arise as a complaint. The cause of the chronic dislocation could be residual ulnar deformity, annular ligament failure, or both [11].

Reducing the radio-ulna joint in Monteggia fracture dislocation is recommended through anatomic reduction and rigid fixation [6,7,11]. If the ulnar deformity is involved in the causing mechanism of radial head dislocation, angulation and lengthening osteotomy of the ulna are required to reduce the radial head [12]. In our case, we ruled out the possibility of a shortened ulna as the cause of radial head subluxation by comparing measurements to the unaffected opposite side as suggested in previous studies, where parameters of normal bilateral radius and ulna should not have any significant difference [[12], [13], [14]].

The annular ligament is part of the lateral collateral ligament complex that originates at the anterior and insertion at the posterior margins of the sigmoid notch of the ulna. It stabilizes the proximal radioulnar joint during pronation and supination [11]. The annular ligament can be reconstructed using autografts or allografts, however, most studies prefer the use of autografts harvested near the elbow area [15,16]. A variation of the Bell Tawse procedure was presented by Marinello et al., where the patients showed regained full range of motion and function in the long-term follow-ups [16]. Reconstruction using cadaveric peroneal graft can be considered, Carlotta et al. performed this procedure due to the limitation in harvesting autograft in the elbow area [17]. Another graft option was shown by Belani et al. who used 4 cm of synthetic graft for annular ligament reconstruction with fixation at the lateral border of the proximal ulna with anchor suture which showed good functional outcome but with distinct erosion at the radial neck in the 2-year follow-up [15].

The original Bell Tawse procedure used a 10 cm × 0.5 cm tricep graft with intact insertion and wrapped the radial head towards the anterior and back to the posterior side, entering a tunnel through the ulna where it then wrapped to the lateral side of the ulna and stitched near the insertion on the posterior side to reconstruct the annular ligament in pediatric patients [10]. Reports regarding the Bell Tawse procedure were mostly done in children [1]. The size decided for the graft needed may not be suitable for adult patients. This was possibly the reason for the inadequate graft length in our case. Adequate tricep tendon graft length with similar measurements was achieved by Marinello et al. possibly because of the detachment of the insertion and fixation of both graft ends to the original annular ligament footprint without the use of bone tunnels [16]. An adult case reported by Carlotta et al. used a longer 12 cm × 0.4 cm cadaveric peroneal tendon graft for the reconstruction and attached it to the medial side, instead of the posterior side, with a suture [17].

Endo-button was used in bicep rupture fixation, anterior cruciate ligament reconstruction, treatment of acromioclavicular dislocation, and many others [18,19]. It hadn't been used in annular ligament reconstruction, especially the Bell Tawse procedure. Suture anchors and sutures are more commonly used in the procedure, with previous reports showing stable elbow and functional range of motion similar to ours [16,20]. We used the endo-button as a cortical fixation for the distal end of the tricep tendon graft of the Bell Tawse procedure to compensate for the short graft. No fixed suture length is necessary between the endo-button and tendon during fixation. This property helps in adding extra length to grafts, especially if graft tension needs to be adjusted or if the graft length is insufficient, as seen in our study [21].

Endo-button fixation has better pullout strength than other fixations, with a low failure rate [21,22]. Worner et al. found that endo-button tendon fixation using OrthoCord suture was significantly lower in rupture rate compared to suture anchor fixation [22]. A cadaveric study by Rutka et al. with bicep tendon grafts showed that screw fixation is more likely to fail than endo-button fixation due to screw pullout from the tunnel [21]. In a comparative study between transosseous suture and endo-button fixation for the repair of distal bicep rupture by Recordon et al., the two techniques were comparable in terms of functional outcome [23]. These studies shows that the use of endo-button compared to other fixation, including suture anchor and screw fixation, is superior in terms of failure rate, with comparable functional outcome [[21], [22], [23]].

Conversely, cortical breakage, suture breakage, and graft rupture can occur in endo-button fixation but with a significantly greater force compared to screw failure (407.78 N and 257.87 N) [21]. Other possible complications include postoperative stiffness with loss of pronation and supination arc due to overtight reconstruction [16]. These potential complications can be avoided with proper adjustment of the tension during reconstruction [16,24]. Another risk to the procedure that needs to be carefully noticed is that endo-button fixation reported by Azoulay et al. showed less rigid fixation in a coracoid bone-block fixation, which may cause displacement to the graft [24]. Our fixation, however, was done to a tendon graft through a bone tunnel, allowing fusion of graft and bone which ensures stability [25]. In our case, at one-year follow-up, no complication was present and the patient reports a satisfying functional outcome. This shows that in the long-term, the use of endo-button in the Bell Tawse can give satisfactory outcome for the management of chronic and recurrent radial head dislocation.

## 4 Conclusion

Radial head dislocations, especially anteriorly, are rarely seen in adults and often neglected in Monteggia fracture-dislocation, resulting in a chronic recurrent condition. Damage to the annular ligament is one of the causes, and reconstruction can be performed using the Bell Tawse procedure. Endo-button hadn't been used in annular ligament reconstruction, especially the Bell Tawse procedure. In our report, we used endo-button cortical fixation to compensate for the inadequate graft length in an adult case. At the one-year follow-up, the patient had no complaints of pain and could do normal activities.

## Consent

Consent was given directly from the patient for this study.

## Ethical approval

This study was approved by our institute's ethical committee.

## Guarantor

All authors; Renaldi Prasetia, Siti Zainab Bani Purwana, Azir Adityo Rahman Dibyosubroto, Yosep A. Tarong, and Raden Andri Primadhi are the Guarantor of this study.

## Funding

There is no external funding.

## CRediT authorship contribution statement

**Renaldi Prasetia:** Conceptualization, Methodology, Resources, Supervision, Writing – review & editing, Project administration. **Siti Zainab Bani Purwana:** Investigation, Data curation, Formal analysis, Writing – original draft. **Azir Adityo Rahman Dibyosubroto:** Investigation, Data curation. **Yosep A. Tarong:** Data curation, Visualization. **Raden Andri Primadhi:** Supervision, Writing – review & editing.

## Declaration of competing interest

There is no conflict of interest in this study.
